# Important steps to improve translation from medical research to health policy

**DOI:** 10.1186/1479-5876-11-33

**Published:** 2013-02-08

**Authors:** Fan Jiang, Jun Zhang, Xiangdong Wang, Xiaoming Shen

**Affiliations:** 1 Department of Developmental and Behavioral Pediatrics, Shanghai Children’s Medical Center, Shanghai Jiao Tong University School of Medicine, Shanghai, China; 2 Ministry of Education-Shanghai Key Laboratory of Children’s Environmental Health, Xinhua Hospital, Shanghai Jiao Tong University School of Medicine, Shanghai, China; 3 Department of Respiratory Medicine, Biomedical Research Center, Shanghai Respiratory Research Institute, Fudan University Medical School, Shanghai, China

## Abstract

Translational medicine entails not only “from-bench-to-bedside” but also preventive medicine. The present article proposes a conceptual framework of translational research from scientific research to health care policy and public health policy. We highlight the importance of translational medicine to bridge between research and policy and share our experience of translating medical research to public health policy in China as well as obstacles and challenges we are facing in the translation process.

## Background

Translational Medicine (TM) has become a fashionable term in medical society and seems important to almost everyone despite that the term is variably defined by different stakeholders. It was described as a “from bench to bedside” pathway in early 1990s when the concept of TM had just emerged. More specifically, it referred to “the transfer of new understandings of disease mechanisms gained in the laboratory into the development of new methods for diagnosis, therapy, and prevention” [1]. Such a definition was commonly accepted and even adapted to categorize types of life-science researchers [2,3].

However, it is being increasingly recognized that this definition is incomplete within the overall TM concept and neglects the importance of health policy. The disconnection between research and health policy was described as “Sound of one hand clapping”, which may have contributed to the scarcity of TM studies on health policy. For instance, we reviewed articles published in two specialty journals in TM: *Journal of Translational Medicine* from January, 2007 to June, 2012 and *Science Translational Medicine* from October, 2009 to June, 2012. Among the 833 published articles, only one focused on government implementation of public policies [4].

Several TM frameworks have been proposed in recent years [5-7]. T3 activities of the policy making in the road map of TM proposed by Dougherty and Conway address “how” health care is delivered [5]. Others emphasized that public health policy should be included into TM framework [6,7]. However, the role of health policy in the TM model has not been clearly illustrated. We emphasize the importance of health policy in achieving the ultimate goal of improving population health here using two examples from China, and discuss obstacles and challenges that we are facing in the translation from medical research to health policy, an integral part of translational medicine.

**Figure 1:**
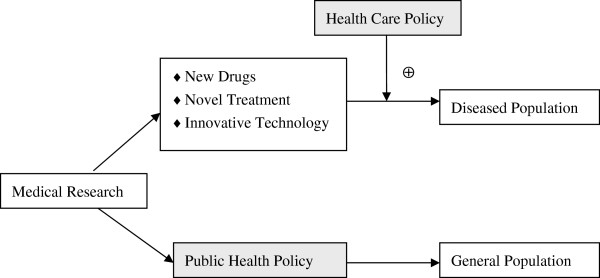
Health policy in translational medicine.

We would like to point out that in our view, health policy here includes *health care policy* and *public health policy*, which achieve their goals through different pathways. Figure 1 illustrates that *health care policy* facilitates deliveries of new drugs, technologies, and therapies to diseased population, while *public health policy* directly impacts on the general population.

### Child lead poisoning

Childhood lead poisoning used to be considered as a problem in industrialized countries but not in China. However, a series of well designed, comprehensive epidemiological and clinical studies on child lead poisoning in Shanghai, China, between 1988 and 1996, demonstrated that lead poisoning was a real threat to Chinese children. Leaded gasoline was found to be the major source of lead. These findings were confirmed by other studies in different locations of the country [8,9], indicating that childhood lead poisoning in China should be prevented.

**Figure 2:**
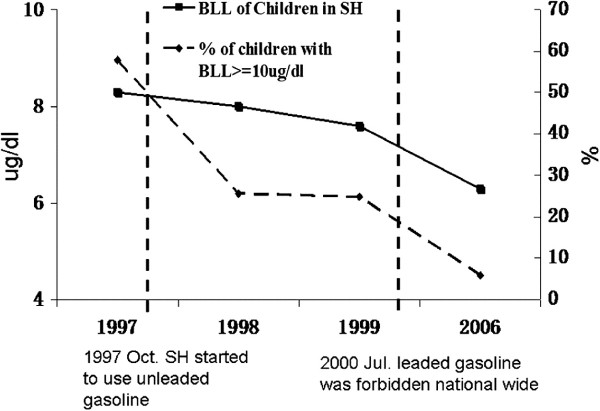
Blood level of lead and prevalence of lead poisoning in Shanghai children (1–5 years old) in relation to local and national policies of banning leaded gasoline.

We advocated the importance of taking an immediate action on the prevention of lead poisoning to the government on numerous occasions, in order to make the real impact on improving child health based on those research findings. After then the government decided to phase out leaded gasoline in Shanghai starting as a pilot project in 1997. The proportion of children with blood lead level above 10 ug/dl, at which WHO recommends public health actions be initiated, decreased from 57.8% in 1997 to 25.7% in 1998 [10]. Leaded gasoline was banned nationwide in July, 2000. Blood lead level in children of Shanghai continued to decline and the prevalence of the lead level above 10 ug/dl decreased to 5.8% by 2006, as shown in Figure 2[11]. It took less than 10 years that average levels of blood lead in the children dropped from 8.5 to 6.5 ug/dl in Shanghai. An estimated gain of 3 points in mean population IQ score was associated with the decline of mean levels of blood leads, based on the calculation according to Canfield’s study result, which showed that a 1 μg/dL increase results in a 1.37 IQ decrease especially when blood lead level is below 10 μg/dL [12]. This gain in IQ also could be calculated to yield an annual economic benefit, for which public health policy of phasing out lead-gasoline is crucial and apparently is not only beneficial to children’s health but to the whole society.

### Sleep insufficiency in school-aged children

An adequate amount of sleep in high quality is important to maintain the health and functioning in the optimal condition. Sleep insufficiency in children and adolescents has been associated with cognitive deficits, mood disturbance and weight gain. Previous studies demonstrated that adolescents could not reliably introspect on the actual level of sleepiness after chronic mild sleep restriction, as compared with young adults, even when the impairment in reaction time was noticed during working memory tasks [13].

**Figure 3:**
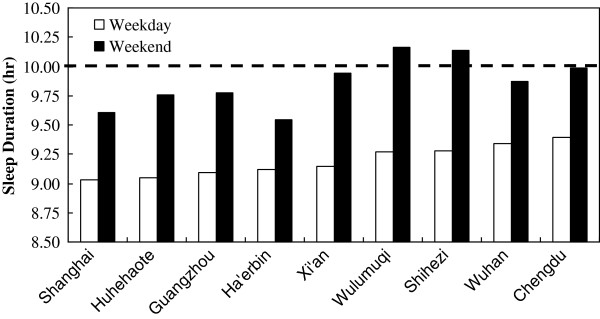
Sleep duration of school-aged children from nine cities in China.

Sleep insufficiency is common in children and adolescents in both developed and developing countries [14,15]. The average duration of sleep per night among American adolescents decreased from 9.1 hr in 1910 [16] to 7.4 hr in 1994 [17]. A recent sleep survey of 1,365 Chinese adolescents at 12–18 years old revealed that the mean duration of sleep was 7.64 hr/night [18]. We conducted a sleep survey with national cluster random sampling of school-aged children in 9 cities in 2005 and found that the average sleep duration in child weekday was significantly shorter than the level of the national recommendation of 10 hr/day. Children in Shanghai had the shortest duration, as shown in Figure 3[19]. School schedule, particularly the start time, was found to be one of influencing factors in the occurrence of sleep insufficiency and daytime sleepiness [19] .

Policy-makers were persuaded to pay close and immediate attention to the sleep insufficiency in children on basis of our epidemiological studies. Shanghai Municipal Education Commission subsequently issued two regulations regarding the sleep intervention which was proposed to adjust school start time. School start time was then changed from 7:15 to 8:15 o’clock for elementary schools and from 7:00 to 8:00 o’clock for middle schools since 2007. We furthermore conducted a validation study in 10 elementary schools in Shanghai that participated in the national sleep survey between 2005 and 2009 to evaluate the effectiveness of the intervention. Our results showed that a 30-minute delay of school start time increased the average sleep duration by 23.4 minutes and decreased the prevalence of daytime sleepiness by 9% [20]. It is worth noting that our studies were conducted in collaboration with Shanghai Education Research Institute affiliated to Shanghai Municipal Education Commission, which facilitated the translation from research to the local government.

### Challenges and opportunities

A number of obstacles and challenges still remain even though some successes in translating medical research into public health policy have been achieved. There are substantial differences in value, need, and culture between research community and policy makers. Researchers, mostly from academic settings, are less motivated to translate medical research into public health policy, since academic promotion and tenure decisions are mainly based on the quantity and quality of publications in scientific journals and the number of funded research grants. Translating clinical and epidemiological studies into public health policy is often neglected, though it is understandable that scientists are expected to do highly sophisticated, cutting-edge studies and to get funding and good publications. China established one of the national highest awards called National Award of Advances in Science and Technology that honors successful translational research with great public health impacts, to encourage more scientists to translate medical research to public health policy.

Another obstacle in translational research is the barrier of communication between researchers and policy makers, as described by Mirvis [21]. It may be difficult for policy makers to understand and have the access to research findings, to meet researchers and discuss the feasibility of translating results of medical research into public health policy, due to different educational and cultural backgrounds. It is our experience that it will be more beneficial if policy makers can be engaged as early as possible and provided with clear and simple messages, which has been proved by our experience. The success of our translational process was attributed to the fact that the staff in the governmental agencies could be involved in the project at the early stage during which they discussed the study at each step of progress. Those research results could be better understood and an appropriate action could be then taken by policy makers accordingly. Public health advocacy is a skill that requires education, training, and practice. Scientific researchers should be highly encouraged to have such skills breaking through one of major barriers, hesitation to approach policy makers. Direct communication between researchers and policy makers will benefit the process of translational medicine from research with the high quality to public health policies.

In conclusion, translational medicine is more than “from bench to beside”. The process from medical research to public health policy is an integral part of translational medicine. An effective mechanism is needed to encourage scientists to conduct studies with high public health impact, engage policy makers as early as possible, and improve public advocacy skills. Hopefully, those efforts will make a great impact in improving population health.

## Competing interests

The authors declare that they have no competing interests.

## Authors’ contribution

This paper was planned by Dr. XMS and he was responsible for the overall conception. Dr. FJ drafted the manuscript. Drs. JZ and XDW were responsible for critical revisions of the manuscript for important intellectual content. All authors read and approved the final manuscript.
